# Congenital laryngeal webs: from diagnosis to surgical outcomes

**DOI:** 10.1016/j.bjorl.2020.06.018

**Published:** 2020-08-03

**Authors:** Melissa Ameloti Gomes Avelino, Débora Bressan Pazinatto, Stela Oliveira Rodrigues, Rebecca Maunsell

**Affiliations:** a Universidade Federal de Goiás (UFG), Departamento de Cirurgia e Serviço de Otorrinolaringologia, Goiânia, GO, Brazil; b Universidade Estadual de Campinas (Unicamp), Departamento de Otorrinolaringologia, Campinas, SP, Brazil

**Keywords:** Child, Congenital laryngeal anomaly, Congenital laryngeal web, Surgical procedure

## Abstract

**Introduction:**

Congenital laryngeal webs are rare, may be associated with other airway abnormalities and be one of many features of microdeletion 22q11. Meticulous evaluation is imperative when deciding which surgical technique to use. The choice of appropriate stenting may be decisive to avoid persistent anterior commissure synechia and poor voice results.

**Objective:**

To report outcomes for endoscopic and open surgical approaches in the treatment of congenital laryngeal webs and the challenges one may encounter while applying the current classification and deciding for the best treatment option.

**Methods:**

Retrospective review of medical and surgical charts for congenital laryngeal webs treated in two tertiary university centers.

**Results:**

Seven patients were included; following Cohen’s classification there were: three type II webs, one of them with an atypical posterior synechia, two type III webs and two type IV webs. Six patients were submitted to laryngotracheal reconstruction and one was treated with an endoscopic approach. Description of precise glottic and subglottic involvement and tailored surgical options are presented. The LT mold® stent was used for long-term stenting that varied between 40 to 60 days. All patients were successfully decannulated with good voice quality and after follow-up of over one year, there were no complications associated with the surgeries.

**Conclusion:**

Congenital laryngeal webs should be suspected and thoroughly evaluated in the presence of neonatal dysphonia and early onset of laryngitis. Otolaryngologists must be familiar with associated lesions and genetic conditions that may be associated to congenital laryngeal webs. Congenital laryngeal webs may be successfully treated at an early age. The correct choice of surgical technique after meticulous evaluation of glottic and subglottic components of the web, presence of concomitant lesions and appropriate stenting, is imperative to avoid persistent scarring and poor voice quality.

## Introduction

Congenital laryngeal webs are rare abnormalities and account for 5% of congenital laryngeal anomalies despite Cohen’s description of a prevalence of one in 1200 at the Children’s Hospital of Los Angeles.^1^ They are considered a failure of recanalization of the primitive larynx that should occur between 8th and 10th weeks of embryogenesis. Other congenital malformations may occur simultaneously, such as congenital subglottic stenosis, trachea-esophageal fistulas and some syndromes.^2^, ^3^ Although a gene has not been identified as the cause of this malformation, genetic investigation and counseling is imperative^3^, ^4^ due to the relatively frequent association with 22q11 microdeletion.

Traditionally laryngeal webs are classified in four subtypes proposed by Cohen.^1^ Type I membranes consist of less than 35% of glottic involvement, are usually thin and do not extend to the subglottic region. Type II webs, although still thin or moderately thick, have a 35%–50% glottic involvement and may present concomitant isolated subglottic stenosis. Type III webs have a 50%–75% glottic involvement, are thick and potentially have a cartilaginous involvement of the adjacent subglottic region. Type IV webs are uniformly thick and involve from 75%–90% of the glottic area with cartilaginous subglottic extension.^1^, ^2^ In types I and II vocal cords are usually visible through the web, while in types III and IV delimitation of vocal cords can be difficult.^1^

Clinical manifestations vary according to the extent of glottic involvement and obstruction. Shorter webs account for dysphonia perceived as a weak cry and may also be the cause of respiratory distress, depending on the degree of obstruction or during upper airway infections. In types III and IV webs, respiratory distress is usually noticeable from birth.^5^, ^6^

Surgical treatment in infants will depend on the extent of the web and symptoms of airway obstruction. In types III and IV webs a tracheostomy may be necessary as an emergency measure to secure the airway during the first days and/or months of life. Due to perioperative risks and technical difficulties of operating on a small airway, definitive surgical treatment is not advised systematically before 6–12 months of age.^7^

The aim of this study is to report outcomes for endoscopic and open surgical approaches in the treatment of congenital laryngeal webs and the challenges one may encounter, both in applying the current classification and deciding for the best treatment option.

## Methods

All children (0–14 years of age) surgically treated for laryngeal webs from the period between 2014 and 2018 were included in the study. Exclusion criteria was follow-up shorter than 6 months. Medical and surgical charts of children treated for laryngeal webs in two tertiary university healthcare centers were described regarding symptoms, classification, associated malformations of the airway, need for tracheostomy, type of surgery performed, type and length of stenting and outcomes for voice and airway symptoms. The study was approved for application in both institutions under the protocol CAAE 17322619.8.0000.5404.

A systematic search of data in the PubMed, Cochrane, LILACS databases was performed to review and compare surgical procedures and outcomes with those described elsewhere.

## Results

Seven patients underwent laryngotracheal reconstruction due to congenital laryngeal webs in two healthcare institutions from 2014 to 2018, with a minimum of 6-month follow up period. The average age at time of surgery was 20 months. The patients’ age ranged from 14 months to 3 years old at the time of surgery.

Clinical presentation, tracheostomy timing, Cohen classification and type of surgery performed can be appreciated in Table 1.

**Table 1:** Reviewed pediatric patients with operated laryngeal web.

	Case 1	Case 2	Case 3	Case 4	Case 5	Case 6	Case 7
Gender	Male	Female	Female	Female	Male	Female	Male
Symptoms	Hoarseness, recurrent laryngitis, biphasic stridor	Neonatal respiratory distress and recurrent laryngitis	Hoarseness, recurrent laryngitis, respiratory distress	Neonatal stridor and respiratory distress	Hoarseness, recurrent laryngitis	Neonatal stridor and respiratory distress	Neonatal stridor and respiratory distress
Age at diagnosis	12 months	11 months	12 months	1 month	4 months	12 months	Few days old
Tracheostomy before treatment	No	Yes	No	Yes	Yes	Yes	Yes
Cohen classification	II	II	Atypical II (posterior web, normal subglottis)	III	III	IV	IV
Age at surgery	14 months	22 months	18 months	36 months	18 months	19 months	18 months
Type of surgery	Endoscopic excision of web (1st procedure)	LTR + AG (DS)	Endoscopic excision of anterior and posterior web + balloon dilation	LTR + AG (DS)	LTR + AG (DS)	LTR + PG (DS)	LTR + APG (DS)
	LTR + AG (DS) (2nd procedure)						
Stenting duration	60 days	60 days	42 days	60 days	60 days	60 days	45 days
22q11.2 microdeletion associated	Yes	Yes	No investigation	Yes	No investigation	No investigation	Yes
Outcome – airway	Decannulated	Decannulated	Decannulated	Decannulated	Decannulated	Decannulated	Decannulated
Time to decannulate after stent removal	At the same time	6 months	21 days	30 days	30 days	At the same time	40 days

LTR, Laryngotracheal Reconstruction; AG, Anterior Graft; APG, Anterior and Posterior Grafts; PG, Posterior Graft; DS, Double Stage.

**Figure 1:**
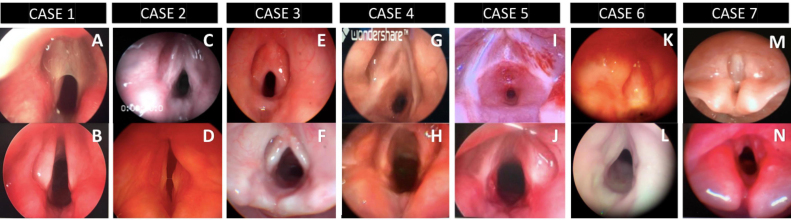
Pre-operative and postoperative (pre op and post op) views of the larynx. Pre-operative views of Cases 1–7 from the top left to right A, C, E, G, I, K and M. Post-operative views of Cases 1–7 from bottom left to right B, D, F, H, J, L and N.

After preoperative mapping with Microlaryngoscopy and Broncoscopy (MLB) under general anesthesia, laryngeal webs were classified according to Cohen as: type II webs in three patients (Fig. 1A – Case 1, C – Case 2 and E – Case 3), type III web in two patients (Fig. 1G – Case 4 and I – Case 5) and type IV webs in two cases (Fig. 1K – Case 6 and M – Case 7). In both institutions, vocal cord spreaders and sizing of the airway with age appropriate tracheal tubes were part of the routine mapping of the airway. One of the type II webs (Fig. 1E – Case 3) presented an atypical interarythenoid synechia without any history of previous intubation. None of the type II webs had a history of previous intubation. Case 1 (Fig. 1A) was a type II laryngeal web, a thin web with no adjacent subglottic extension but elliptical subglottic area sized as grade II subglottic stenosis. Case 5 (Fig. 1I) had additional acquired subglottic stenosis related to a single recent intubation that ultimately led to a tracheostomy.

Almost all types III and IV cases (Cases 4, 6 and 7) underwent tracheostomies during the first days of life due to respiratory distress. Case 5 had a tracheostomy performed at 14 months of age after intubation following an episode of “laryngitis”. The main complaint of the three type II (Cases 1, 2 and 3) cases was weak voice and recurrent laryngiti,s and one of them required a tracheostomy prior to the laryngotracheal reconstruction due to the severity of obstructive episodes and logistic of timing of reconstructive surgery (Case 2).

Four patients (57%) had characteristic phenotype features and diagnosis of 22q11 microdeletion was confirmed after referral to the geneticist (Cases 1, 2, 4 and 7). The other three patients did not complete genetic investigation, although none of them present phenotype features.

**Figure 2:**
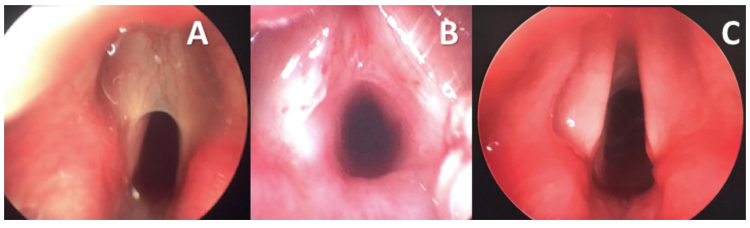
Case 1 — type II laryngeal web. (A) Intraoperative view before web incision; (B) subglottic stenosis revealed after web incision; (C) post-operative endoscopic view.

Six patients underwent laryngotracheal reconstruction (LTR) and one was treated with an endoscopic approach. Case 1 presented a very thin type II web (Figs. 1A and 2 A) that was initially divided endoscopically with cold knife instrumentation. The airway was sized using an endotracheal tube and revealed a low-grade II Subglottic Stenosis (SGS) (Fig. 2B). An attempt was made to extubate the patient without further treatment of the Grade II SGS, but this failed and laryngotracheal reconstruction with an anterior graft was performed 48 h later (Fig. 2C). The type II web treated through an endoscopic approach (Case 3) presented an atypical posterior supraglottic/glottic component. Following endoscopic section of the web and the “posterior synechia” the airway was sized and revealed a normal size subglottis. For this reason, an option was made to stent the airway with a Long-Term stent (LT-mold) placed endoscopically and then proceed only with a tracheostomy.

In all cases the cartilaginous subglottic component of the web or stenosis was assessed and submucosal remodeling with a diamond burr or cold knife was performed prior to cartilaginous graft expansion as described by Trey et al.^2^

**Figure 3:**
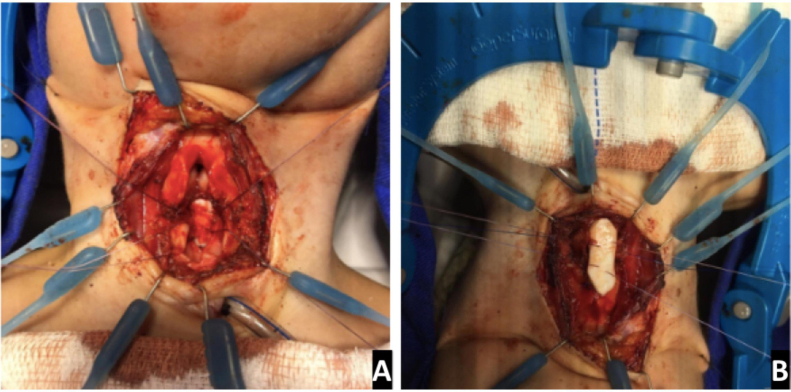
Grafting. LTR anterior and posterior graft being fixed to the expanded cricoid cartilage (Case 7) (A) posterior graft (B) anterior graft.

Cartilage rib grafts were used to expand the airway (Fig. 3). The decision on anterior and /or posterior grafts depended both on endoscopic evaluation and sizing of the airway as well as on the anatomical configuration of the cricoid and subglottic area intraoperatively.

**Figure 4:**
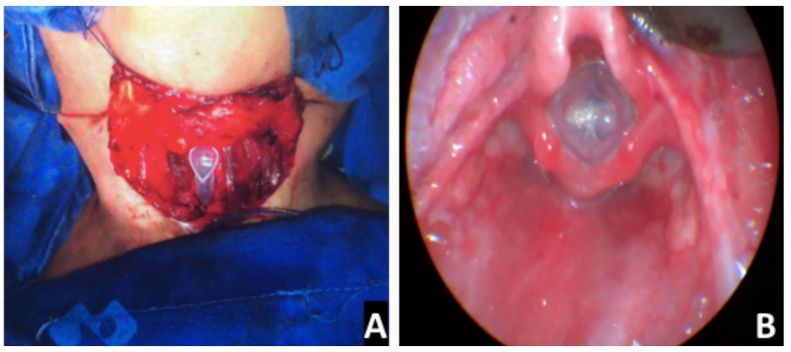
Stenting. (A) Intraoperative view of an inserted stent (Case 4); (B), endoscopic view of the LT-Mold (Case 3).

Monnier’s LT-mold® stents were used in all procedures (Fig. 4). Duration of stenting varied from 42 to 60 days with a mean duration of 55 days. Decannulation was possible on average 43 days after stent removal.

None of the patients presented any complications and at follow- up there were no complaints of recurrent respiratory episodes or laryngitis, dyspnea or dysphonia. Follow-up time was over one year, and all patients were successfully decannulated with good voice quality that was described by all parents as “better than before surgery”. Voices were considered to be understandable and satisfactory for communication both with family and strangers despite speech and language issues particularly in the cases with confirmed 22q11.2 microdeletion. Pre- and post-operative view of laryngoscopy can be appreciated in Fig. 1.

A systematic search of available data on the subject of treatment options for laryngeal webs in children identified only five articles after revision, with specific inclusion strategies to avoid disparities and selection bias as listed in Table 2.

**Table 2:** Database search results regarding reports on Laryngeal web surgical treatment. Search performed in May 2020.

Database	Search strategy	Articles found in first search	Articles found in second search^a^	Articles considered^b^	Articles included^c^
PUBMED	(laryngeal diseases/congenital) AND (laryngeal diseases/surgery) (laryngeal membrane) OR (laryngeal atresia) OR (laryngeal web) OR (laryngeal webs) AND (children)	232	44	10	5
LILACS	(laryngeal) AND (congenital malformations)	5	−	0	0

a The second search was performed without MESH terms to increase the number of articles reviewed.

b Only articles specifically regarding surgical treatment of congenital laryngeal webs in the last 10 years.

c Criteria used for exclusion: articles including other laryngeal pathologies; articles with adult patients, articles that did not focus on surgical treatment or articles that reported a single case.

## Discussion

Dysphonia at birth should always raise suspicion towards laryngeal webs. All seven patients reported dysphonia at birth. Although not all presented with neonatal stridor, those that did not experienced episodes of laryngitis during the first six months. Persistent dysphonia and recurrent laryngitis or, laryngitis occurring at an atypical age should be investigated and referred to an experienced otolaryngologist for evaluation.

Newborns with dysphonia and/or respiratory distress should undergo an awake nasofibroscopy which will be conclusive for the diagnosis.^2^ Nevertheless, Microlaryngoscopy and Broncoscopy (MLB) under general anesthesia is imperative to evaluate extension of the web to the subglottic area, size the airway with appropriate endotracheal tubes and search for other concomitant airway anomalies.^4^ This is important to avoid “blind” intubation and further scarring of an abnormal larynx as seen in Case 5.

In the present report one of the cases presented an associated airway malformation (Table 1). As originally described by Cohen, isolated subglottic stenosis can be encountered^1^ in type II laryngeal webs, as occurred in Case 1. Interarythenoid synechia with no history of intubation as found in Case 2 has not been described and this illustrates how variable congenital webs can be, reinforcing the need for meticulous examination.

Cohen’s classification is very useful and shows very good correlation with symptoms: with types I and II webs being less symptomatic and types III and IV more symptomatic.^1^ Nevertheless, particularly in type II webs where both endoscopic and open approaches may be good options, there may be a wide variation in presentation as illustrated by these cases. Type II webs may be very thin with good delimitation of the vocal cords, a bit thicker, with subglottic extension or not or, even, associated with isolated SGS (as in Case 1) and in each case a different approach may be considered. Restoring the best voice and airway may be achieved by meticulous submucosal correction of cartilaginous malformation, airway expansion with a cartilage graft, mucosal preservation and use of a smooth stent that can sustain the “V” – shaped anterior commissure. One should not rely only on this classification to predict the best surgical approach and, particularly in type II web surgeries must be tailored to each patient on a case to case basis.

The incidence of 22q11 microdeletion in patients presenting laryngeal webs is reported to be as high as 65%.^2^, ^8^ Lawlor et al. in the largest case series published to this day over an observation period of 22 years reported 10 out of 16 congenital webs (62%) with 22q11 microdeletion. This genetic disorder, also known as Di George Syndrome or Shprintzen Syndrome, is caused by microdeletion of 22q11 which is tested through Fluorescent In Situ Hybridization (FISH) or direct sequencing.^5^, ^6^, ^8^ Not all patients with 22q11 microdeletion will present the same or the full range of malformations such as cardiac alterations therefore genetic consultation is imperative. This is the most frequent microdeletion occurring in humans and knowledge on its different presentations is increasing.^9^ Frequency of velopalatal insufficiency, immunodeficiency, delayed speech, and learning issues are particularly high and important to be addressed at an early stage. Four (57%) of the reported patients tested positive and three patients have not completed investigation but do not present phenotypical features.

Endoscopic surgery and web incision with either cold knife instruments or laser, is a very popular treatment choice, especially if there is no subglottic extension.^10^ Nevertheless, there is a potential for synechia formation at the anterior commissure.^5^, ^6^ Some authors suggest the use of keels in endoscopic cases to avoid anterior synechia. The use of keels should be seen with caution in small children since respiratory insufficiency and scarring of the glottis area can occur from the use of these. In contrast to adults the use of a keel in children requires a tracheostomy to secure the airway. One of the reported patients initially underwent endoscopic surgery, one with simple incision of the web followed by LTR after failed extubation. Another was treated with a single endoscopic procedure that consisted of web incision, balloon dilation and LT-Mold endoscopic stenting. Mytomicin C was not used in any of these cases.

In all the current cases Monnier’s LT-mold® was used to stent the glottic area. Its triangular shape at the glottis level helps support the anterior commissure and expands the subglottic area at the same time during reepithelization.^2^ Although other materials and stents may be adapted, particularly in the small airway, use of a smooth stent that perfectly fits the shape of the infants’ larynx should help prevent secondary granulation tissue and scarring.^2^ Surgeons should be very cautious adapting stents and particularly keels in small airways since airway obstruction may occur even without visible granulation tissue formation. There is no consensus as to the time the stent should be left in place.^11^ Stenting time in the reported surgeries varied from 42 to 60 days. Unfortunately, the LT-Mold is currently not commercially available.

Successful treatment should result in a patent functional airway and a satisfactory voice quality. Patients with type III and IV laryngeal webs are at greater risk for poor voice quality and residual subglottic stenosis due to extensive glottic involvement and associated subglottic malformation.^2^

Voice quality described in this report was subjective and longer follow-up is needed to conclude that vocal needs are still satisfactory in the long run up until the adolescent and adult age.

Systematic search of the literature revealed five articles that specifically report surgical treatment for laryngeal webs and outcomes. With the exception of the group from Lausanne reported by Trey et al.,^2^ case reports are quite heterogeneous. The Lausanne group reports more severe types III and IV laryngeal webs. Rodriguez et al. reported eight cases, one of which was a type I web with no surgical treatment needed and another type III web for which no surgical treatment was described except a tracheostomy.^5^, ^7^

Goudy et al. in 2010 reported the largest series of 18 cases over a 25-year period.^12^ Lawlor et al. recently reported 37 cases but only 16 congenital in a 22-year period and over half the cases were grade I laryngeal webs, both congenital and acquired.^9^ Surgical experience in both these series probably varied greatly considering the time spam over which the cases were treated, the number of different surgeons and the evolving experience over the years. Despite the relatively large patient numbers these two studies had reported, Lawlor et al. treated only two grade IV webs^9^ and Goudy et al. only one grade IV web.^12^ In the current study the time period was considerably shorter, only two surgeons performed all surgeries and none of the cases had been treated previously. Despite most patients having Grade III and IV webs number of procedures and time to decannulation was considerably smaller. In recent years understanding of these lesions has shown that grade III and IV laryngeal webs are best treated with open approaches.^2^, ^12^ This may explain why no complications were observed and results were so satisfactory with patients decannulated after a maximum of 6 months post operatively. Lawlor et al. described 8 congenital Grade III and 2 grade IV laryngeal webs and only one open surgery being indicating before any other endoscopic attempt.^9^ These authors found a recurrence rate of 45% leading to multiple procedures in grade III and IV laryngeal webs, while in the current series patients were asymptomatic after one procedure. The initial choice for an endoscopic approach in the treatment of these high-grade laryngeal webs may have contributed to further scarring and a suboptimal treatment of the airway.

Trey et al. is the only study that specifically describes voice outcomes, also in a subjective manner.^2^ Their group describes a better voice postoperatively in 91% of their 14 cases with over 50% having a good or mildly dysphonic voice. This group also used the LT mold as a stent and their relatively good voice results despite treating severe laryngeal webs may also the use of a more anatomically favorable stent alongside with meticulous surgical technique.

Identification of congenital malformation of the cricoid is very important prior and intraoperatively in LTR’s. Submucosal remodeling of the cricoid cartilage in these cases may allow for meticulous reconstruction of the subglottic area as has been described by the Lausanne group.^2^ The cases reported in this study followed the principles of mucosal approximation after cartilage remodeling and atraumatic stenting and this might have accounted for less scarring and favorable voice results.

Although this is a limited number of cases, congenital laryngeal webs are very rare and surgical reports are scarce, particularly regarding postoperative results. Objective voice results are challenging in small children, nevertheless, parental reports of postoperative satisfactory voices in a previously dysphonic child cannot be underestimated.

Future studies should focus on long-term follow up of voice quality and voice performance in children treated for laryngeal webs, particularly for type II webs with little airway compromise that might eventually be postponed. Comparison of results in larger homogenous groups would be ideal; this may be achieved in the future with multicenter groups following precise classification and outcome protocols.

## Conclusion

Congenital laryngeal web should be suspected and thoroughly evaluated in the presence of neonatal dysphonia and early onset of laryngitis. Otolaryngologists must be familiar with associated lesions and genetic conditions that may be associated to congenital laryngeal webs. Congenital laryngeal webs are successfully treated at an early age. Choice of surgical technique after meticulous evaluation of glottic and subglottic components of the web, presence of concomitant lesions and appropriate stenting is imperative to avoid persistent scarring and poor voice quality.

## Conflicts of interest

The authors declare no conflicts of interest.

## References

[1] Cohen S.R. (1985). Congenital glottic webs in children. A retrospective review of 51 patients. Ann Otol Rhinol Laryngol Suppl.

[2] Trey L.A., Lambercy K., Monnier P., Sandu K. (2016). Management of severe congenital laryngeal webs — a 12 year review. Int J Pediatr Otorhinolaryngol.

[3] Izadi F., Delarestaghi M.M., Memari F., Mohseni R., Pousti B., Mir P. (2010). The butterfly procedure: a new technique and review of the literature for treating anterior laryngeal webs. J Voice.

[4] Nicollas R., Triglia J.M. (2008). The anterior laryngeal webs. Otolaryngol Clin North Am.

[5] Rodríguez H.A., Cuestas G., Zanetta A. (2013). Dysphonia in children due to congenital laryngeal web. Case series. Arch Argent Pediatr.

[6] Benmansour N., Remacle M., Matar N., Lawson G., Bachy V., Vorst S.V. (2012). Endoscopic treatment of anterior glottic webs according to Lichtenberger technique and results on 18 patients. Eur Arch Otolaryngol.

[7] Sztanó B., Torkos A., Róvó L. (2010). The combined endoscopic management of congenital laryngeal web. Int J Pediatr Otorhinolaryngol.

[8] Parkes W.J., Propst E.J. (2016). Advances in the diagnosis, management, and treatment of neonates with laryngeal disorders. Semin Fetal Neonatal Med.

[9] Lawlor C.M., Dombrowski N.D., Nuss R.C., Rahbar R., Choi S.S. (2020). Laryngeal web in the pediatric population: evaluation and management. Otolaryngol Head Neck Surg.

[10] Xiao Y., Wang J., Han D., Ma L., Ye J., Xu W. (2014). Vocal cord mucosal flap for the treatment of acquired anterior laryngeal web. Chin Med J (Eng).

[11] Bajaj Y., Cochrane L.A., Jephson C.G., Wyatt M.E., Bailey C.M., Albert D.M. (2012). Laryngotracheal reconstruction and cricotracheal resection in children: recent experience at Great Ormond Street Hospital. Int J Pediatr Otorhinolaryngol.

[12] Goudy S., Bauman N., Manaligod J., Smith R.J. (2010). Congenital laryngeal webs: surgical course and outcomes. Ann Otol Rhinol Laryngol.

